# Interactions Between Brain ^18^F-FDG PET Metabolism and Hemodynamic Parameters at Different Ages of Life: Results From a Prospective Cross-Sectional Study

**DOI:** 10.3389/fnagi.2022.908063

**Published:** 2022-06-28

**Authors:** Gaétan Zimmermann, Laure Joly, Pauline Schoepfer, Matthieu Doyen, Veronique Roch, Rachel Grignon, Paolo Salvi, Pierre-Yves Marie, Athanase Benetos, Antoine Verger

**Affiliations:** ^1^CHRU Nancy, Department of Nuclear Medicine and Nancyclotep Imaging Platform, Université de Lorraine, Nancy, France; ^2^CHRU Nancy, Geriatric Department, Université de Lorraine, Nancy, France; ^3^INSERM, DCAC, Université de Lorraine, Vandoeuvre Les Nancy, France; ^4^IADI, INSERM U1254, Université de Lorraine, Nancy, France; ^5^Cardiology Unit, Instituto Auxologico Italiano, IRCCS, Milan, Italy

**Keywords:** brain ^18^F-FDG-PET, hemodynamics, cerebral aging, central arterial pressure, arterial stiffness

## Abstract

Brain ^18^F-FDG PET imaging is useful to characterize accelerated brain aging at a pre-symptomatic stage. This study aims to examine the interactions between brain glycolytic metabolism and hemodynamic parameters in different age groups.

**Methods**: A total of 72 patients (from 23 to 88 years of age, 38 women) without any cerebral diseases but with available cardiac, arterial peripheral, and central blood pressure measurements as well as arterial stiffness parameters obtained from brachial pressure and applanation tonometry and a brain ^18^F-FDG PET scan were prospectively included into this study. Quantitative voxel-to-voxel analyses were carried out to test for negative associations between brain glycolytic metabolism and individual hemodynamic parameters (p-voxel of <0.001 for the whole population and <0.005 for age groups).

**Results**: The heart rate parameter of the whole population showed the most extensive associations with brain metabolism (15,857 mm^3^, T-score: 5.1), predominantly affecting the frontal and temporal regions (69% of the volume). Heart rate for the younger age group, systolic and pulse pressure for the 41–60-year-old group, and diastolic pressure for the older group were most extensively associated with brain metabolism and mainly involved the fronto-temporal lobes (respective involvement of 52.8%, 60.9%, and 65.5%) which are also the regions implicated in accelerated brain aging.

**Conclusion**: This cross-sectional prospective study identified extensive associations between cerebral metabolism and hemodynamic parameters, indicating common aging mechanisms. Heart rate throughout adult life, systolic and pulse pressure parameters around middle age, and diastolic pressure parameters in older patients, suggest the existence of potentially therapeutic targets to prevent accelerated brain aging.

## Introduction

Associations between hemodynamic parameters and accelerated cerebral aging are now widely established, in particular chronic high blood pressure, which is the most important risk factor for cognitive decline in older adults (Gąsecki et al., 2013). In our previous study, we reported that impaired cerebral metabolism was significantly associated with a central pulse pressure greater than 50 mmHg in cognitively asymptomatic hypertensive older adults, highlighting the importance of measuring central hemodynamic parameters when investigating brain metabolism (Verger et al., 2015). Resting heart rate, a marker of cardiac autonomic function, is related to cardiovascular disease and atherosclerosis through sympathetic overactivity and pulsatile shear stress. Moreover, autonomic regulation seems to have consequences on cognitive processes (Forte et al., 2019). Among the hemodynamic parameters, those targeting arterial stiffness, a typical marker of arterial aging, can be appreciated directly by measuring the pulse wave velocity (PWV; Salvi et al., 2004; Laurent et al., 2006), which is associated with cerebral small vessel disease and an accelerated decline of cognitive functions even in very old adults (Kearney-Schwartz et al., 2009; Singer et al., 2014; Watfa et al., 2015; Pase et al., 2016).

^18^F-FluoroDesoxyGlucose Positron Emission Tomography (^18^F-FDG PET), which allows to investigate brain glycolytic metabolism (Guedj et al., 2022), helps to characterize accelerated brain aging at the pre-symptomatic stage. Previous studies have suggested that elevated peripheral arterial pressure levels (Langbaum et al., 2012) and even more so elevated central arterial pressure levels (Verger et al., 2015) lead to local reductions in cerebral metabolism in cognitively non-affected patients. These data illustrate the impact of hemodynamic parameters on brain function, at the preclinical remodeling stage. In addition, a large epidemiological study correlated elevated central arterial pressure levels with an increased risk of cardiac and/or cerebral stroke (Roman et al., 2009). To the best of our knowledge, there are however no currently available data that show the impact of hemodynamic parameters, including central arterial pressure, on the cerebral metabolism of participants at different ages of life.

This cross-sectional prospective study, therefore, aims to investigate associations between brain glycolytic metabolism, determined by ^18^F-FDG PET imaging, and hemodynamic parameters, as a function of age, in a population with no cerebral pathologies.

## Materials and Methods

### Population and Study Design

One hundred participants referred to the University Hospital of Nancy (France) for an ^18^F-FDG PET between August 2018 and November 2020, but with no oncological or neurological indications, were prospectively included in the PACTEP (Central Arterial Pressure and Positron Emission Tomography) study (Number Clinical Trials.gov: NCT03345290). Participants were age-stratified, at their time of inclusion into the study, and consisted of a 25% distribution of 18- to 40-year-old participants, 25% of 41- to 60-year-old participants, and 50% over 60-year-old participants. Pregnant women, minors, persons under judicial protection, participants with a history of psychiatric or neurological cerebral pathologies, or patients taking psychotropic drug treatments, participants who received chemotherapy or encephalic radiotherapy, and participants with atrial fibrillation or severe carotid stenosis were not included. Main indications for the ^18^F-FDG PET were: (1) an initial assessment or follow-up of inflammatory diseases (44%) such as granulomatosis (18%), vasculitis (8%), and others (18%; i.e., lupus, Sjögren’s syndrome, polymyalgia rheumatic, and spondyloarthropathy), with all these PET indications retained as covariates in our voxel-to-voxel quantitative analyses; (2) an etiological assessment of hyperthermia, decline in general condition (17%); (3) an initial assessment of cancer or suspected cancer, before any treatment (20%, including 13% in thoracic oncology); (4) an assessment of adenopathy or investigation of a hematological syndrome prior to any treatment (11%); and (5) an initial assessment or follow-up of an infectious pathology (8%), mainly alveolar echinococcosis.

On the day of the ^18^F-FDG PET scan patients, included in the study, initially performed a battery of neuropsychological tests: Mini-Mental State Examination (MMSE; Folstein et al., 1975), the Frontal Assessment Battery (FAB; Dubois et al., 2000), and the Mini International Neuropsychologic Interview (MINI; Sheehan et al., 1998). Any patients diagnosed with a cognitive disorder (MMSE < 27, FAB < 15) or a major depressive syndrome (as defined by the MINI-test) were subsequently excluded from the study.

This cross-sectional study was approved by the local ethics committee (CPP accreditation N°2018/26). All participants signed an informed consent form before enrollment. This research complied with the principles of the Declaration of Helsinki. This clinical trial was reported in the Clinical Trials database (NCT03345290).

### Hemodynamic Parameters

Before their ^18^F-FDG PET scan and after resting 15 min in the supine position, participants were set up in a quiet room and three peripheral arterial pressure measurements were taken with an automated monitor (DINAMAP v100, General Electric^®^) and averaged to improve representativity (peripheral systolic blood pressure, pSBP and peripheral diastolic blood pressure, pDBP). Peripheral pulse pressure (pPP) was obtained by subtracting pDBP from pSBP.

Central pulse parameters were measured by two experienced physicians (PSc, LJ) using applanation tonometry (Pulsepen DiaTecne^®^, San Donato Milanese, Italy; Salvi et al., 2004). All results were expressed as an average of 10 measurements obtained from 10 cardiac cycles. The central systolic blood pressure (cSBP), central diastolic blood pressure (cDBP) parameters were derived from the analysis of the carotid pulse wave. The central pulse pressure (cPP) was determined by subtracting cDBP from cSBP, and the heart rate (HR) was established from the ECG. Carotid-femoral pulse wave velocity (cf-PWV) was obtained by applying the tonometer to the carotid and femoral arteries as previously reported (Van Bortel et al., 2012).

### ^18^F-FDG PET Acquisition and Data Analyses

Brain PET acquisitions were performed on a digital camera (Vereos, Philips^®^), after a neurosensory rest of 45 min following the intravenous injection of 3.5 MBq of ^18^F-FDG. All participants had fasted at least 6 h prior to receiving the injection and had blood glucose levels < 10 mmol/L. Acquisitions were recorded over a 15 min single bed position and all brain PET scans were reconstructed with the OSEM iterative reconstruction algorithm, as used in clinical practice, and corrected for PSF (Point Spread Function), attenuation, random coincidences, and scatter. Reconstruction parameters were based on two iterations of 10 subsets, displayed in a 256 × 256 matrix with 1 × 1 × 1 mm^3^ (Doyen et al., 2021). The acquisition was then followed by the whole-body ^18^F-FDG PET for which the patient was referred. All brain PET images were carefully visually reviewed by nuclear physician specialists to exclude any patients with potential brain abnormalities.

^18^F-FDG PET brain images were processed using the Statistical Parametric Mapping, SPM 12, software (running on Matlab 2020b (MathWorks Inc., Sherborn, MA). An initial spatial normalization step was carried out with the low-dose scanner which is required for attenuation correction using the adaptive template registered to the Montreal National Institute (MNI) space. Transformations applied to the CT images of each patient were then applied to the co-registered PET images. The size of the scan voxels was set to 1 × 1 × 1 mm^3^. Intensity was normalized to the pons, a reference region with a glycolytic metabolism that is only weakly influenced by age (Verger et al., 2021) or by the presence of neurodegenerative pathologies (Minoshima et al., 1995). All images were subsequently smoothed with a 4 mm full-width at half-maximum Gaussian-filter to limit individual variations in gyral anatomy. Due to the advanced age of our population (>50% of patients over 60 years of age), partial volume effect corrections were applied, based on the Müller-Gärtner method (Müller-Gärtner et al., 1992) using the PETPVE12 toolkit (Greve et al., 2016). For this, a segmentation of white and gray matter was carried out on CT scans using the SPM tools after a visual analysis to check the accuracy of this segmentation.

Statistical analyses were performed at group-level and voxel-to-voxel using linear regression models of previously detailed hemodynamic parameters with an exclusive white matter mask. Covariates evaluated included age, considered as a continuous variable as well as sex, education level, the presence/absence of anti-hypertensive therapy, and the presence/absence of an inflammatory disease that may affect brain metabolism (Kalpouzos et al., 2009; Lee et al., 2012; Kim et al., 2015). These analyzes were initially performed on the entire population using a p-voxel value of 0.001 and subsequently on the three separate age groups (18–40, 41–60, and >60 years) using a less-restrictive p-voxel value of 0.005. All reported clusters were corrected for cluster volume based on Monte-Carlo simulations to avoid type II errors (Lieberman and Cunningham, 2009). Only negative associations between brain metabolism and hemodynamic parameters were retained. Specific cluster locations in the MNI space were obtained by using the SPM xjView toolbox^1^. For linear regression analyses, only clusters with a cumulative volume over 10 cm^3^ (i.e., at least 1% of the total encephalic volume) were considered as significant. Images were visually inspected at different stages of the pre-processing procedure to ensure the quality and convergence of the different methods applied (MD).

### Statistical Analysis

Categorical variables are expressed as percentages and continuous variables as medians and interquartile ranges. Because variables were not normally distributed, Chi-2 and Kruskal-Wallis tests were performed to compare categorical and continuous variables, respectively. A p-value < 0.05 was considered to be significant. All tests were performed in SPSS (SPSS Statistics for Windows, Version 20.0. Armonk, NY: IBM Corp). The statistical analysis for SPM is detailed in the previous section.

## Results

### Population

**Figure 1:**
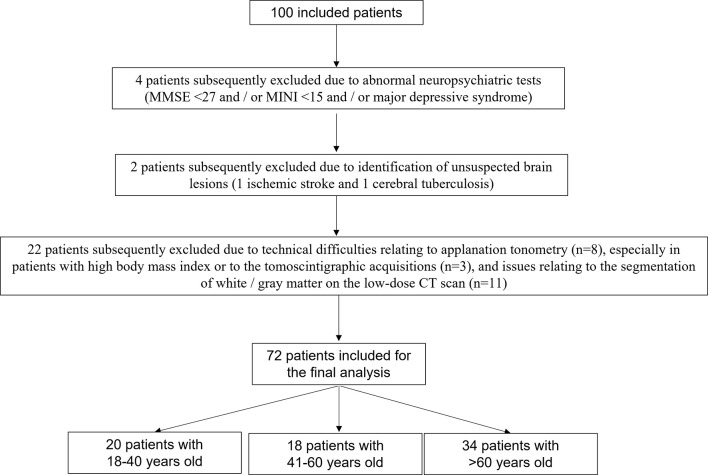
Flowchart of patients included in the final analysis.

The SPM analysis was finally performed for 72 patients. Of the 100 candidates initially identified for inclusion in the study, 28 were subsequently excluded due to: (i) abnormal neuropsychiatric tests (four participants with an MMSE < 27 and/or MINI < 15 and/or major depressive syndrome), (ii) the identification of unsuspected brain lesions (one ischemic stroke and one cerebral tuberculosis), (iii) technical difficulties relating to applanation tonometry (eight participants), especially in patients with high body mass index (mean body mass index of these participants of 31.6 ± 6.2 kg.m^−2^) (Joly et al., 2010) or to the tomoscintigraphic acquisitions (three participants), and (iv) issues relating to the segmentation of white/gray matter on the low-dose CT scan which impeded corrections for the partial volume effect (11 participants). A flowchart for the final population used for the analyses is displayed in Figure 1.

Patients ranged from 23 to 88 years of age (mean age = 55.4 years ± 17.1) and were stratified into three age groups (18–40 years, *n* = 20; 41–60 years, *n* = 18; > 60 years, *n* = 34). There were no significant differences between the three age groups in terms of the sex distribution and the presence/absence of an inflammatory disease. As expected, there were significant differences in blood pressure (pSBP, cSBP, pPP) and arterial stiffness (cf-PWV) between the younger and older patients. Clinical and hemodynamic characteristics of the whole population and of the three age groups are given in Table 1.

**Table 1:** Population characteristics.

**Group**	**Whole population (*n* = 72)**	**18–40 years old (*n* = 20)**	**41–60 years old (*n* = 18)**	**>60 years old (*n* = 34)**	***p* values**
**Population**					
Age (years)	57.3 (38.4; 70.2)	32.0 (28.9; 37.5)	53.8 (47.8; 56.2)	70.6 (65.0; 73.8)	<0.001*^¥^*
Sex (Female)	38 (52.8%)	9 (45.0%)	13 (72.2%)	16 (47.1%)	0.160
Educational level					0.001*
None	12 (16.7%)	0 (0.0%)	0 (0.0%)	12 (35.3%)	
Primary school	23 (31.9%)	6 (30.0%)	5 (27.8%)	12 (35.3%)	
High school	15 (20.8%)	4 (20.0%)	7 (38.9%)	4 (11.8%)	
College	22 (30.6%)	10 (50.0%)	6 (33.3%)	6 (17.6%)	
Anti-hypertensive treatment	27 (37.5%)	2 (10.0%)	4 (22.2%)	21 (61.8%)	<0.001*
Inflammatory disease	34 (47.2%)	10 (50.0%)	5 (27.8%)	19 (55.9%)	0.148
					
HR (bpm)	67.0 (58.0; 77.0)	61.5 (55.0; 69.8)	67.5 (59.0; 72.3)	68.0 (60.5; 77.0)	0.269
					
pSBP (mmHg)	124.5 (116.0; 133.3)	116.5 (111.8; 122.5)	126.0 (116.8; 130.8)	131.5 (121.0; 141.8)	<0.001*
pDBP (mmHg)	71.0 (64.8; 79.3)	66.0 (63.8; 72.0)	74.5 (64.0; 79.3)	72.0 (66.0; 80.8)	0.130
pPP (mmHg)	53.0 (47.0; 61.0)	49.5 (43.8; 52.3)	54.5 (46.5; 62.0)	55.0 (52.3; 64.3)	0.015*
					
cSBP (mmHg)	121.0 (113.0; 132.3)	113.5 (109.8; 119.3)	111.3 (105.3; 121.0)	127.0 (117.5; 137.3)	0.004*
cDBP (mmHg)	71.0 (65.0; 78.0)	69.0 (64.3; 72.3)	86.0 (77.6; 91.3)	73.0 (65.5; 78.8)	0.190
cPP (mmHg)	48.0 (43.0; 63.0)	45.0 (40.5; 52.3)	49.5 (44.3; 65.3)	52.5 (43.3; 65.3)	0.122
					
cf-PWV (m/s)	9.3 (7.9; 12.1)	8.1 (7.4; 9.2)	8.5 (7.8; 10.5)	11.3 (8.5; 13.3)	0.001*

p values for comparisons between the three age subgroups; ^¥^significant differences between the three groups, *significant differences between the 18–40 year and the >60-year subgroups. HR, heart rate; pSBP, peripheral systolic blood pressure; pDBP, peripheral diastolic blood pressure; pPP, peripheral pulse pressure; cSBP, central systolic blood pressure; cDBP, central diastolic blood pressure; cPP, central pulse pressure; cf-PWV, carotid-femoral pulse wave velocity.

### SPM Analyses of the Whole Population

**Figure 2:**
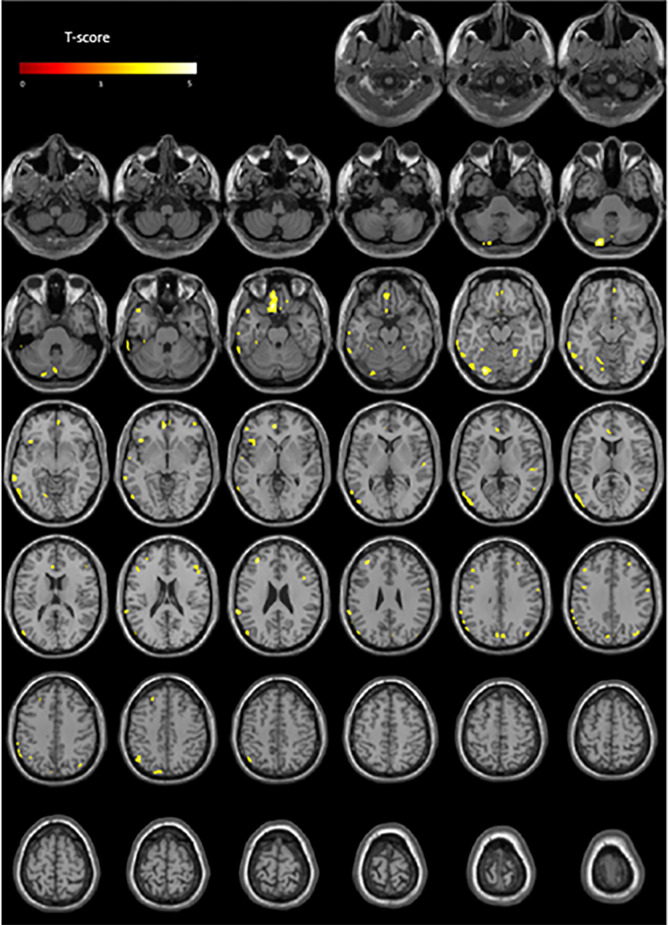
Anatomical localizations of significant brain metabolic clusters associated with HR (negative association) in the whole population (*p* < 0.001, uncorrected) projected onto two-dimensional slices of T1-weighted MRIs (from the base to the top of the skull).

Table 2 summarizes analyses of the whole population. The HR parameter showed the most extensive interactions with cerebral metabolism (37 clusters, cumulative volume of clusters: 15,857 mm^3^, T-score peak: 5.1) specifically with the frontal (35.8% of cumulative volume) and temporal lobe (33.2% of cumulative volume) regions. The T-maps projected onto two-dimensional slices of axial T1-weighted MRIs are given in Figure 2 and the spatial locations of abnormalities are detailed in the Supplementary Material.

**Table 2:** Results of the quantitative voxel-to-voxel analyses for the negative linear regression analyses between hemodynamic parameters and brain glycolytic metabolism on the whole population (p-voxel = 0.001, uncorrected, corrected for cluster volume).

**Parameters**	**NC**	**Volume (mm^3^)**	**T score peak**
HR	37	**15,857**	5.1
pSBP	0	0	0.0
pDBP	3	548	3.9
pPP	0	0	0.0
cSBP	1	100	4.3
cDBP	3	487	4.3
cPP	0	0	0.0
cf-PWV	8	3,447	5.3

*In bold: cumulative cluster volumes, considered as significant for influencing brain metabolism (>10 cm^3^). HR, heart rate; pSBP, peripheral systolic blood pressure; pDBP, peripheral diastolic blood pressure; pPP, peripheral pulse pressure; cSBP, central systolic blood pressure; cDBP, central diastolic blood pressure; cPP, central pulse pressure; cf-PWV, carotid-femoral pulse wave velocity*.

### SPM Analyses Per Age Group

We then evaluated the relative associations of each hemodynamic parameter with cerebral metabolism within each of the three age groups.

In the youngest age group, HR showed the most extensive associations with cerebral metabolism (29 clusters, cumulative volume of the clusters: 12,032 mm^3^, T-score peak: 10.5) with fronto-temporal predominant localizations (52.8% of the cumulative volume of the clusters).

In the 41- to 60-year-old patient group, systolic and pulse pressure parameters exhibited the most extensive associations with cerebral metabolism (cPP, cSBP, pPP, pSBP). This effect was particularly prominent for the central hemodynamic parameters: cPP (61 clusters, cumulative volume: 33,418 mm^3^; T-score peak: 14.9) and cSBP (49 clusters, cumulative volume: 24,597 mm^3^, T-score peak: 8.6), and to a lesser extent for the peripheral parameters: pPP (47 clusters, cumulative volume: 22,186 mm^3^; T-score peak: 8.7) and pSBP (34 clusters, cumulative volume: 16,231 mm^3^; T-score peak: 9.8). Spatial localization analyses showed diffuse metabolic abnormalities related to these parameters, with fronto-temporal predominance in particular for cPP and for cSBP (respectively 60.9% and 42.1% of the total of clusters identified).

**Figure 3:**
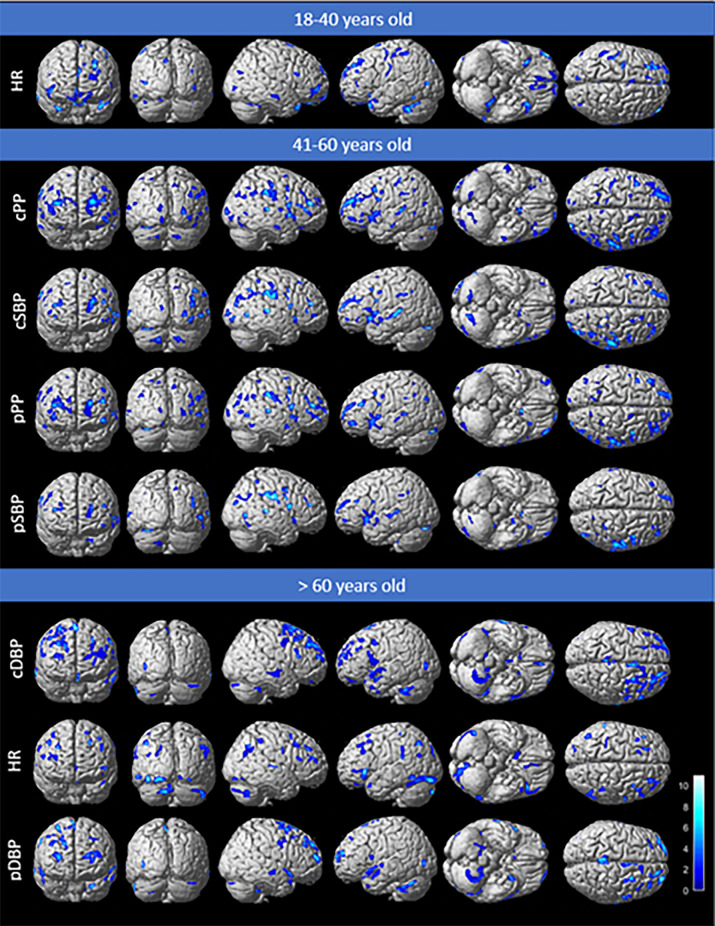
Anatomical localization of clusters associated with parameters that have a significant impact on brain metabolism with negative associations (*p* < 0.005, uncorrected), projected onto 3D volume-rendered images, spatially normalized, and smoothed into the standard SPM template. Parameters classified according to the cumulative significant cluster volumes for the three age groups.

In addition to associations between cerebral metabolism and HR in the oldest age group, cerebral metabolism showed the most extensive associations with the cDBP parameter (26 clusters, cumulative volume: 20,728 mm^3^, T-score peak: 5.7). As depicted in Figure 3, these associations predominantly involved the frontal and the temporal cortex: 65.5% of cumulative volume of the significant clusters were localized in the frontal or temporal lobe for cDBP.

Analyses of all parameters for the different age groups are given in Table 3, T-maps projected onto 3D volume-rendered images are shown in Figure 3 and the spatial location of abnormalities is detailed in the Supplementary Material.

**Table 3:** Results of the quantitative voxel-to-voxel analyses for the linear regression analyses between hemodynamic parameters and brain glycolytic metabolism on the three age groups (*p*-voxel = 0.005, uncorrected, corrected for the cluster volume).

Groups	18–40 years old			41–60 years old			>60 years old		
Parameters	NC	Volume (mm^3^)	T score peak	NC	Volume (mm^3^)	T score peak	NC	Volume (mm^3^)	T score peak
HR	29	**12,032**	10.5	9	2,267	6.3	32	**17,122**	5.2
pSBP	10	3,251	6.5	34	**16,231**	9.8	7	9,750	6.6
pDBP	15	4,334	6.5	1	147	4.5	26	**15,514**	5.4
pPP	2	1,180	4.5	47	**22,186**	8.7	2	2,916	4.7
cSBP	16	6,174	6.6	49	**24,597**	8.6	11	6,537	5.3
cDBP	16	5,569	6.9	0	0	0.0	26	**20,728**	5.7
cPP	14	7,121	6.0	61	**33,418**	14.9	2	438	4.3
cf_PWV	21	6,689	7.8	20	5,396	7.1	1	233	3.5

*In bold: cumulative cluster volumes considered as significant for influencing brain metabolism (differential volume > 10 cm^3^). NC, numbers of clusters; volume, cumulative volume of clusters; HR, heart rate; pSBP, peripheral systolic blood pressure; pDBP, peripheral diastolic blood pressure; pPP, peripheral pulse pressure; cSBP, central systolic blood pressure; cDBP, central diastolic blood pressure; cPP, central pulse pressure; cf-PWV, carotid-femoral pulse wave velocity*.

## Discussion

Our results demonstrate the existence of associations between hemodynamic parameters and cerebral glycolytic metabolism, with HR exhibiting the most extensive interactions with cerebral metabolism in the whole population. Interestingly, systolic and pulse pressure parameters showed the most extensive associations involving the 41-to-60-year age group whereas diastolic pressure was associated with cerebral metabolism in older participants, suggesting that these parameters may represent potential therapeutic targets. These differences among age groups could be explained by physiological hemodynamics modifications related to aging. The effect of hypertension on cognitive impairment, mainly related to systolic and pulse pressure parameters, has been reported in midlife subjects (Knopman et al., 2001). In older subjects, a continuously high level of pressure related to the diastolic level of pressure impacts consistently the brain metabolism, since diastolic compliance decreases with age (Wolsk et al., 2017).

In terms of the whole population, the number of associations of anomalous brain metabolism with hemodynamic parameters was particularly extensive for HR, suggesting an interaction of heart function and brain metabolism which may point to a common pre-symptomatic aging mechanism. To the best of our knowledge, very few studies have to date investigated the impact of resting HR on brain and cognitive decline. The European guidelines identify an HR of greater than 80 bpm as a parameter which impacts cardiovascular risk in hypertensive patients (Williams et al., 2018), since HR has been shown to be a determinant of accelerated progression of arterial stiffness in hypertensive patients (Benetos et al., 2002). Moreover, a high HR is an independent predictor of cognitive decline in patients at high cardiovascular risk (Böhm et al., 2015). This HR effect was also identified in the younger and older age groups of our age subgroup analysis, confirming the premise that HR influences brain metabolism throughout life. It is interesting that our study associated HR with fronto-temporal hypometabolism because fronto-temporal hypometabolism is a hallmark of accelerated brain aging (Verger et al., 2015).

Interactions between brain metabolism and hemodynamic parameters need to be investigated at all stages of life to identify potential targets for therapy. The most extensive associations in younger patients were obtained for HR. This parameter has previously been shown to influence brain metabolism in younger participants. Our data suggest that reducing the HR may decelerate the risk of developing brain abnormalities. One way to reduce HR is to practice a regular aerobic physical activity which has been demonstrated to impart positive effects on cognitive functions in young and middle-aged healthy participants (Cox et al., 2016). Several other studies have shown a positive impact of physical activity on executive functions (Kamijo and Takeda, 2009, 2010), which are localized to the frontal cortex, the region predominantly affected in our voxel-wise analyses (35.5% of the cumulative cluster volume).

In our middle age group, systolic and pulse pressures were the parameters that were most extensively associated with cerebral metabolism, suggestive of hypertension during middle adulthood causing pre-symptomatic brain remodeling, which may be predictive of future cognitive function. These data are consistent with several longitudinal studies which showed that midlife arterial hypertension is a risk factor for cognitive decline and dementia (Launer et al., 2000; Knopman et al., 2001). For peripheral pressure parameters, our results are consistent with the Langbaum et al. study which showed that systolic and pulse pressure are associated with frontal and temporal hypometabolism determined by ^18^F-FDG PET in middle-aged participants (Langbaum et al., 2012). It is also important to underline that central pressure (cPP, cSBP) parameter interactions involved more extensive brain volumes than peripherical parameter (pPP, pSBP) associations, suggesting that data obtained from carotid applanation tonometry are more representative of vascular stresses exerted on the brain. This is consistent with our previous work in older adults with hypertension (Verger et al., 2015). Although the role of reducing blood pressure to prevent the incidence of dementia is still controversial the latest meta-analysis of a large number of interventional trials confirms a significant effect of antihypertensive treatment in older adults (Hughes et al., 2020).

For older participants, cDBP exhibited the most extensive associations with brain metabolism (20.7 cm^3^). Several studies have shown significant associations between high pDBP values and cognitive decline in a middle-aged population (Tsivgoulis et al., 2009) or between high pDBP values and the progression of white matter lesions on MRI in older adults (McNeil et al., 2018), but to the best of our knowledge, no data are currently available for diastolic central parameters even if diastolic peripheral and central pressure values are close to each other. The pulse wave velocity, albeit associated with brain metabolism in older adults, was not an extensive association. This may be explained by 40% of our older adult population being non-hypertensive, and all of our hypertensive participants receiving successful antihypertensive treatments.

From a physio-pathological perspective, associations between hemodynamic parameters and brain metabolism were predominantly localized to the fronto-temporal regions which may correspond to the site of accelerated, vascular origin, cerebral aging. Indeed, hypertension—a risk factor for accelerated brain aging- is predominantly associated with disorders in the frontal and striatal regions and an impairment of executive functions (Buckner, 2004), which is consistent with our data. The potential impact of hemodynamic parameters measured in our study may be related to cerebral small vessel disease. Indeed, MRI hypersignals in white matter—which are evidence of small vessel disease—have been shown to be associated with a significant decrease in gray matter metabolism in older hypertensive adults, specifically involving the frontal cortex (Chetouani et al., 2017).

Our study has several limitations relating to its monocentric nature and its lack of longitudinal follow-up data. In addition, for ethical reasons, the patients included in this study presented with extracerebral pathologies, which motivated the examination. This may have influenced the results, especially for systemic pathologies such as inflammatory diseases, which may have subclinical repercussions on brain imaging. This potentially confounding effect was nevertheless considered in our analyses. By the same token, only a minority of our population was hypertensive (37.5%) and all hypertensive participants were receiving anti-hypertensive treatments, but this is quite representative of the proportion of hypertensive patients in France, based on the latest available epidemiological data (SPF, 2020).

To conclude, we report extensive associations between hemodynamic parameters, specifically HR, and cerebral metabolism throughout adult life. This suggests that brain metabolism and heart function may share common pre-symptomatic aging mechanisms. While systolic and pulse pressure parameters were more prominently involved around middle age, the number of interactions with diastolic pressure parameters were more extensive in older patients. These data suggest the presence of potentially useful therapeutic targets which may be exploited, during specific periods of life, to prevent accelerated brain aging.

## Data Availability Statement

The raw data supporting the conclusions of this article will be made available by the authors, without undue reservation.

## Ethics Statement

The studies involving human participants were reviewed and approved by the ethics committee of CHRU of Nancy (CPP accreditation N°2018/26). The patients/participants provided their written informed consent to participate in this study.

## Author Contributions

AV, LJ, and VR: conceptualization. PSc, GZ, and RG: collection of clinical data. PSc, LJ, and PSa: measurements/analysis of haemodynamic parameters. RG: data curation. GZ, AV, and MD: SPM analysis. GZ, AV, LJ, AB, P-YM, and PSa: redaction—review and editing. All authors contributed to the article and approved the submitted version.

## Conflict of Interest

The authors declare that the research was conducted in the absence of any commercial or financial relationships that could be construed as a potential conflict of interest.

## Publisher’s Note

All claims expressed in this article are solely those of the authors and do not necessarily represent those of their affiliated organizations, or those of the publisher, the editors and the reviewers. Any product that may be evaluated in this article, or claim that may be made by its manufacturer, is not guaranteed or endorsed by the publisher.

## Footnotes

^1^ http://www.alivelearn.net/xjview

## Abbreviations

HR: heart rate
cPP: central pulse pressure
cSBP: central systolic blood pressure
pPP: peripheral pulse pressure
pSBP: peripheral systolic blood pressure
cDBP: central diastolic blood pressure
pDBP: peripheral diastolic blood pressure.
