# Supplementary material for: Interactions Between Brain 18F-FDG PET Metabolism and Hemodynamic Parameters at Different Ages of Life: Results From a Prospective Cross-Sectional Study
Source: Front Aging Neurosci. 2022 Jun 28;14:908063. doi: 10.3389/fnagi.2022.908063 (PMC9273887; doi:10.3389/fnagi.2022.908063)
Supplement: Supplementary file 1 [file Table_1.DOCX]

Supplementary Material

**Table 1.** Clusters identified by the voxel-to-voxel analyses for the linear regression analyses between **heart rate** and brain glycolytic metabolism in the whole population (p-voxel= 0.001, FWE uncorrected, corrected for the cluster volume).

| **Localization** | | | **size** | **T** | **x** | **y** | **z** |
| --- | --- | --- | --- | --- | --- | --- | --- |
| **Left cerebrum** | | |  |  |  |  |  |
|  | Frontal | |  |  |  |  |  |
|  |  | Medial orbital gyrus | 2401 | 5.1 | -8 | 26 | -25 |
|  |  | Anterior cingulate cortex, pregenual | 730 | 4.9 | -4 | 46 | -2 |
|  |  | Inferior frontal gyrus pars orbitalis | 584 | 4.6 | -42 | 23 | -3 |
|  |  | Middle frontal gyrus | 403 | 5.0 | -33 | 43 | 25 |
|  |  | Middle frontal gyrus | 171 | 3.9 | -44 | 29 | 35 |
|  |  | Inferior frontal gyrus, triangular part | 149 | 4.1 | -50 | 41 | 0 |
|  |  | Superior frontal gyrus | 106 | 3.6 | -23 | 35 | 43 |
|  |  | Precentral gyrus | 101 | 3.7 | -46 | 1 | 34 |
|  |  | Inferior frontal gyrus, triangular part | 92 | 3.6 | -47 | 31 | 19 |
|  | Parietal | |  |  |  |  |  |
|  |  | Supramarginal gyrus | 680 | 4.5 | -66 | -39 | 22 |
|  |  | Angular gyrus | 423 | 4.7 | -45 | -64 | 46 |
|  | Temporal | |  |  |  |  |  |
|  |  | Middle temporal gyrus | 2189 | 4.3 | -68 | -38 | -7 |
|  |  | Middle temporal gyrus | 1714 | 4.0 | -49 | -76 | 25 |
|  |  | Temporal pole: middle temporal gyrus | 237 | 4.1 | -45 | 18 | -27 |
|  |  | Fusiform gyrus | 195 | 3.9 | -29 | -45 | -17 |
|  |  | Inferior temporal gyrus | 133 | 3.6 | -62 | -20 | -24 |
|  |  | Superior temporal gyrus | 91 | 3.8 | -56 | -5 | -2 |
|  | Occipital | |  |  |  |  |  |
|  |  | Lingual gyrus | 906 | 4.8 | -25 | -80 | -16 |
|  |  | Lingual gyrus | 367 | 4.0 | -20 | -63 | -11 |
|  |  | Inferior occipital gyrus | 288 | 3.8 | -48 | -74 | -14 |
|  |  | Superior occipital gyrus | 236 | 3.6 | -9 | -84 | 43 |
|  |  | Cuneus | 205 | 3.9 | -5 | -78 | 33 |
| **Right cerebrum** | | |  |  |  |  |  |
|  | Frontal | |  |  |  |  |  |
|  |  | Middle frontal gyrus | 334 | 3.9 | 49 | 34 | 19 |
|  |  | Middle frontal gyrus | 146 | 4.0 | 45 | 49 | -3 |
|  |  | Inferior frontal gyrus, triangular part | 140 | 3.8 | 42 | 15 | 26 |
|  |  | Middle frontal gyrus | 136 | 4.4 | 30 | 39 | 34 |
|  |  | Gyrus rectus | 96 | 3.7 | 6 | 21 | -25 |
|  |  | Medial orbital gyrus | 89 | 4.0 | 14 | 33 | -26 |
|  | Parietal | |  |  |  |  |  |
|  |  | Postcentral gyrus | 164 | 4.0 | 63 | -4 | 31 |
|  | Temporal | |  |  |  |  |  |
|  |  | Fusiform gyrus | 295 | 4.1 | 24 | -52 | -16 |
|  |  | Superior temporal gyrus | 204 | 4.0 | 54 | -24 | 7 |
|  |  | Inferior temporal gyrus | 109 | 3.5 | 52 | -66 | -12 |
|  |  | Middle temporal gyrus | 90 | 3.5 | 49 | -58 | 14 |
|  | Occipital | |  |  |  |  |  |
|  |  | Middle occipital gyrus | 322 | 3.8 | 39 | -77 | 36 |
|  |  | Cuneus | 220 | 3.9 | 5 | -79 | 33 |
| **Cerebellum** | | |  |  |  |  |  |
|  | Left hemisphere | |  |  |  |  |  |
|  |  | Crus II of cerebellar hemisphere | 909 | 4.7 | -18 | -88 | -35 |
|  |  | Crus II of cerebellar hemisphere | 202 | 3.7 | 1 | -80 | -33 |

Size: cluster size (mm3); T: T score of peak; x. y. z: MNI coordinates of peak

**Table 2.** Clusters identified by the voxel-to-voxel analyses for the linear regression analyses between **heart rate** and brain glycolytic metabolism in the 18-40-year group (p-voxel= 0.005, FWE uncorrected, corrected for the cluster volume).

| **Localization** | | | **size** | **T** | **x** | **y** | **z** |
| --- | --- | --- | --- | --- | --- | --- | --- |
| **Left cerebrum** | | |  |  |  |  |  |
|  | Frontal | |  |  |  |  |  |
|  |  | Superior frontal gyrus | 1030 | 5.5 | -28 | 50 | 37 |
|  |  | Precentral gyrus | 552 | 5.1 | -29 | -8 | 49 |
|  |  | Superior frontal gyrus, medial | 518 | 10.5 | -2 | 60 | 30 |
|  |  | Middle frontal gyrus | 172 | 4.5 | -33 | 42 | 28 |
|  |  | Supplementary motor area | 166 | 3.8 | -3 | 9 | 71 |
|  |  | Superior frontal gyrus | 127 | 4.3 | -12 | 33 | 40 |
|  | Parietal | |  |  |  |  |  |
|  |  | Inferior parietal gyrus | 388 | 4.2 | -51 | -40 | 59 |
|  |  | Postcentral gyrus | 346 | 5.2 | -41 | -23 | 58 |
|  |  | Postcentral gyrus | 288 | 4.4 | -49 | -12 | 27 |
|  | Temporal | |  |  |  |  |  |
|  |  | Temporal pole: superior temporal gyrus | 853 | 5.6 | -37 | 22 | -31 |
|  |  | Inferior temporal gyrus | 185 | 4.3 | -63 | -54 | -16 |
|  |  | Temporal pole: superior temporal gyrus | 152 | 4.5 | -37 | 12 | -23 |
|  |  | Temporal pole: superior temporal gyrus | 150 | 3.8 | -47 | 21 | -12 |
|  | Occipital | |  |  |  |  |  |
|  |  | Superior occipital gyrus | 199 | 5.0 | -8 | -83 | 43 |
|  |  | Middle occipital gyrus | 303 | 4.5 | -45 | -77 | 6 |
| **Right cerebrum** | | |  |  |  |  |  |
|  | Frontal | |  |  |  |  |  |
|  |  | Middle frontal gyrus | 628 | 7.9 | 47 | 53 | -4 |
|  |  | Superior frontal gyrus, medial orbital | 418 | 5.1 | 5 | 70 | -11 |
|  |  | Inferior frontal gyrus, triangular part | 228 | 4.2 | 45 | 19 | 23 |
|  |  | Superior frontal gyrus, medial orbital | 223 | 5.3 | 4 | 56 | -7 |
|  |  | Anterior cingulate cortex, supracallosal | 212 | 4.3 | 4 | 36 | 19 |
|  | Parietal | |  |  |  |  |  |
|  |  | Middle cingulate & paracingulate gyri | 255 | 3.7 | 2 | -50 | 33 |
|  |  | Precuneus | 218 | 5.6 | 14 | -57 | 28 |
|  | Temporal | |  |  |  |  |  |
|  |  | Middle temporal gyrus | 530 | 6.1 | 34 | 18 | -36 |
|  |  | Middle temporal gyrus | 206 | 4.4 | 69 | -18 | -15 |
|  | Occipital | |  |  |  |  |  |
|  |  | Inferior occipital gyrus | 166 | 4.0 | 40 | -88 | -3 |
|  |  | Middle occipital gyrus | 129 | 3.6 | 36 | -78 | 25 |
| **Cerebellum** | | |  |  |  |  |  |
|  | Left cerebellum | |  |  |  |  |  |
|  |  | Crus I of cerebellar hemisphere | 2357 | 5.4 | -36 | -44 | -38 |
|  |  | Crus I of cerebellar hemisphere | 581 | 3.8 | -41 | -61 | -28 |
|  | Right cerebellum | |  |  |  |  |  |
|  |  | Lobule VI of cerebellar hemisphere | 452 | 4.4 | 36 | -62 | -28 |

*Size: cluster size (mm3); T: T score of peak; x. y. z: MNI coordinates of peak*

**Table 3.** Clusters identified by the voxel-to-voxel analyses for the linear regression analyses between **central pulse pressure** and brain glycolytic metabolism in the 41-60-year group (p-voxel= 0.005, FWE uncorrected, corrected for the cluster volume).

| **Localization** | | | **size** | **T** | **x** | **y** | **z** |
| --- | --- | --- | --- | --- | --- | --- | --- |
| **Left cerebrum** | | |  |  |  |  |  |
|  | Frontal | |  |  |  |  |  |
|  |  | Superior frontal gyrus | 3360 | 11.4 | -24 | 51 | 23 |
|  |  | Middle frontal gyrus | 603 | 7.3 | -42 | 12 | 43 |
|  |  | Middle frontal gyrus | 429 | 5.3 | -42 | 53 | -6 |
|  |  | Inferior frontal gyrus. opercular part | 359 | 5.8 | -53 | 7 | 24 |
|  |  | Inferior frontal gyrus pars orbitalis | 170 | 4.2 | -49 | 36 | -12 |
|  |  | Precentral gyrus | 139 | 4.9 | -53 | 1 | 37 |
|  |  | Superior frontal gyrus | 124 | 6.1 | -23 | 2 | 61 |
|  |  | Inferior frontal gyrus, triangular part | 106 | 4.6 | -50 | 32 | 1 |
|  | Parietal | |  |  |  |  |  |
|  |  | Middle cingulate & paracingulate gyri | 1488 | 7.0 | -9 | -42 | 37 |
|  |  | Postcentral gyrus | 509 | 5.3 | -40 | -30 | 59 |
|  |  | Inferior parietal gyrus | 255 | 4.3 | -48 | -41 | 35 |
|  |  | Precuneus | 175 | 3.9 | -8 | -58 | 36 |
|  |  | Middle cingulate & paracingulate gyri | 134 | 4.0 | -9 | 20 | 33 |
|  |  | Precuneus | 129 | 7.5 | -15 | -36 | 66 |
|  |  | Angular gyrus | 111 | 4.3 | -40 | -56 | 41 |
|  |  | Precuneus | 107 | 4.9 | -6 | -52 | 11 |
|  |  | Superior parietal gyrus | 99 | 5.1 | -25 | -51 | 59 |
|  | Temporal | |  |  |  |  |  |
|  |  | Middle temporal gyrus | 800 | 5.7 | -59 | -31 | 7 |
|  |  | Rolandic operculum | 545 | 6.4 | -55 | 10 | 2 |
|  |  | Fusiform gyrus | 202 | 5.4 | -26 | -50 | -15 |
|  |  | Middle temporal gyrus | 199 | 4.5 | -59 | -18 | -19 |
|  |  | Middle temporal gyrus | 105 | 4.6 | -47 | -55 | 2 |
|  | Insula | |  |  |  |  |  |
|  |  | Insula | 1931 | 9.9 | -35 | 19 | -4 |
|  | Basal ganglia | |  |  |  |  |  |
|  |  | Nucleus accumbens | 565 | 5.3 | -9 | 9 | -8 |
|  |  | Lenticular nucleus, Putamen | 416 | 9.3 | 33 | 1 | 3 |
|  |  | Lenticular nucleus, Putamen | 350 | 6.6 | -24 | 1 | -1 |
|  | occipital | |  |  |  |  |  |
|  |  | Calcarine fissure and surrounding cortex | 307 | 4.6 | -8 | -93 | -11 |
|  |  | Middle occipital gyrus | 114 | 4.3 | -12 | -103 | 8 |
|  |  | Cuneus | 112 | 4.0 | -10 | -93 | 13 |
| **Right cerebrum** | | |  |  |  |  |  |
|  | Frontal | |  |  |  |  |  |
|  |  | Anterior cingulate cortex. supracallosal | 5784 | 8.3 | 5 | 21 | 25 |
|  |  | Superior frontal gyrus | 1533 | 11.7 | 30 | 43 | 20 |
|  |  | Inferior frontal gyrus, triangular part | 1079 | 5.6 | 47 | 35 | 9 |
|  |  | Inferior frontal gyrus, opercular part | 780 | 5.8 | 53 | 8 | 16 |
|  |  | Precentral gyrus | 274 | 6.9 | 45 | 4 | 44 |
|  |  | Supplementary motor area | 159 | 4.6 | 10 | 11 | 53 |
|  |  | Precentral gyrus | 151 | 6.2 | 58 | 4 | 28 |
|  |  | Superior frontal gyrus | 132 | 3.9 | 17 | 57 | 27 |
|  |  | Supplementary motor area | 127 | 6.0 | 15 | 5 | 66 |
|  |  | Paracentral lobule | 107 | 4.4 | 4 | -44 | 59 |
|  |  | Superior frontal gyrus | 102 | 4.0 | 22 | 52 | 26 |
|  |  | Posterior orbital gyrus | 94 | 3.8 | 32 | 26 | -22 |
|  | Parietal | |  |  |  |  |  |
|  |  | Supramarginal gyrus | 2047 | 14.9 | 57 | -32 | 39 |
|  |  | Postcentral gyrus | 584 | 6.4 | 46 | -22 | 50 |
|  |  | Angular gyrus | 367 | 4.6 | 61 | -54 | 34 |
|  |  | Angular gyrus | 231 | 5.4 | 50 | -69 | 29 |
|  |  | Superior parietal gyrus | 145 | 4.7 | 17 | -67 | 47 |
|  |  | Angular gyrus | 141 | 4.1 | 48 | -59 | 44 |
|  |  | Inferior parietal gyrus | 116 | 4.4 | 45 | -52 | 37 |
|  | Temporal | |  |  |  |  |  |
|  |  | Rolandic operculum | 1740 | 5.5 | 52 | 8 | -2 |
|  |  | Superior temporal gyrus | 558 | 5.8 | 59 | -28 | 9 |
|  |  | Middle temporal gyrus | 299 | 4.4 | 69 | -12 | -21 |
|  |  | Heschl’s gyrus | 156 | 5.6 | 61 | -6 | 6 |
|  |  | Middle temporal gyrus | 123 | 3.9 | 58 | -64 | 1 |
|  | Occipital | |  |  |  |  |  |
|  |  | Middle occipital gyrus | 418 | 14.9 | 29 | -99 | 7 |
|  |  | Middle occipital gyrus | 259 | 5.9 | 41 | -78 | 34 |
|  |  | Inferior occipital gyrus | 234 | 5.2 | 40 | -90 | -5 |
|  |  | Lingual gyrus | 191 | 4.6 | 25 | -56 | -3 |
| **Cerebellum** | | |  |  |  |  |  |
|  | Left hemisphere | |  |  |  |  |  |
|  |  | Crus I of cerebellar hemisphere | 519 | 5.3 | -30 | -86 | -23 |
|  |  | Crus II of cerebellar hemisphere | 250 | 3.9 | -15 | -77 | -39 |
|  | Right hemisphere | |  |  |  |  |  |
|  |  | Crus I of cerebellar hemisphere | 428 | 4.7 | 20 | -72 | -37 |
|  |  | Lobule VI of cerebellar hemisphere | 347 | 4.7 | 26 | -70 | -21 |

*Size: cluster size (mm3); T: T score of peak; x. y. z: MNI coordinates of peak*

**Table 4.** Clusters identified by the voxel-to-voxel analyses for the linear regression analyses between **central systolic blood pressure** and brain glycolytic metabolism in the 41-60-year group (p-voxel= 0.005, FWE uncorrected, corrected for the cluster volume).

| **Localization** | | | **size** | **T** | **x** | **y** | **z** |
| --- | --- | --- | --- | --- | --- | --- | --- |
| **Left cerebrum** | | |  |  |  |  |  |
|  | Frontal | |  |  |  |  |  |
|  |  | Anterior cingulate cortex, supracallosal | 2814 | 5.2 | 1 | 21 | 23 |
|  |  | Superior frontal gyrus | 1209 | 7.4 | -21 | 63 | 14 |
|  |  | Inferior frontal gyrus, triangular part | 280 | 4.6 | -42 | 34 | 24 |
|  |  | Middle frontal gyrus | 240 | 5.0 | -40 | 15 | 46 |
|  |  | Precentral gyrus | 207 | 5.1 | -54 | 6 | 24 |
|  |  | Precentral gyrus | 145 | 4.6 | -54 | 1 | 38 |
|  |  | Supplementary motor area | 103 | 4.7 | -10 | 6 | 64 |
|  |  | Inferior frontal gyrus pars orbitalis | 99 | 3.4 | -49 | 39 | -9 |
|  |  | Middle frontal gyrus | 97 | 4.5 | -41 | 56 | -6 |
|  | Parietal | |  |  |  |  |  |
|  |  | Middle cingulate & paracingulate gyri | 1532 | 7.7 | -12 | -38 | 38 |
|  |  | Inferior parietal gyrus | 379 | 7.6 | -47 | -40 | 35 |
|  |  | Inferior parietal gyrus | 125 | 4.4 | -35 | -54 | 37 |
|  |  | Precuneus | 118 | 3.8 | -10 | -56 | 36 |
|  |  | Precuneus | 104 | 4.6 | -7 | -52 | 10 |
|  |  | Middle cingulate & paracingulate gyri | 103 | 3.6 | -8 | 3 | 38 |
|  | Temporal | |  |  |  |  |  |
|  |  | Middle temporal gyrus | 928 | 7.0 | -66 | -27 | 2 |
|  |  | Rolandic operculum | 680 | 6.9 | -60 | 3 | 5 |
|  |  | Superior temporal gyrus | 201 | 5.4 | -58 | -2 | -9 |
|  |  | Hippocampus | 122 | 4.4 | -31 | -10 | -15 |
|  | Insula | |  |  |  |  |  |
|  |  | Insula | 2860 | 7.7 | -35 | 14 | -7 |
|  | Basal ganglia | |  |  |  |  |  |
|  |  | Nucleus accumbens | 263 | 4.3 | -5 | 10 | -3 |
|  |  | Lenticular nucleus. Putamen | 105 | 4.6 | -24 | 1 | -2 |
|  | Occipital | |  |  |  |  |  |
|  |  | Cuneus | 165 | 3.7 | -11 | -92 | 14 |
| **Right cerebrum** | | |  |  |  |  |  |
|  | Frontal | |  |  |  |  |  |
|  |  | Superior frontal gyrus | 665 | 6.7 | 29 | 43 | 20 |
|  |  | Inferior frontal gyrus, opercular part | 290 | 4.5 | 55 | 9 | 16 |
|  |  | Inferior frontal gyrus, triangular part | 166 | 4.5 | 48 | 35 | 8 |
|  |  | Precentral gyrus | 157 | 4.6 | 46 | 4 | 43 |
|  |  | Precentral gyrus | 141 | 5.3 | 56 | 4 | 30 |
|  |  | Middle frontal gyrus | 128 | 4.8 | 39 | 50 | -1 |
|  |  | Precentral gyrus | 115 | 5.1 | 17 | -32 | 66 |
|  |  | Superior frontal gyrus | 114 | 4.0 | 20 | 17 | 55 |
|  |  | Anterior cingulate cortex, pregenual | 108 | 3.8 | 7 | 46 | 15 |
|  |  | Superior frontal gyrus | 102 | 4.1 | 11 | -65 | 47 |
|  | Parietal | |  |  |  |  |  |
|  |  | Supramarginal gyrus | 2311 | 8.6 | 58 | -26 | 32 |
|  |  | Postcentral gyrus | 318 | 4.5 | 46 | -22 | 51 |
|  |  | Postcentral gyrus | 157 | 5.3 | 29 | -34 | 50 |
|  |  | Superior parietal gyrus | 156 | 5.5 | 31 | -47 | 58 |
|  |  | Angular gyrus | 142 | 3.9 | 59 | -51 | 35 |
|  |  | Precuneus | 102 | 5.4 | 18 | 26 | 55 |
|  | Temporal | |  |  |  |  |  |
|  |  | Superior temporal gyrus | 646 | 6.1 | 65 | -28 | 7 |
|  |  | Middle temporal gyrus | 333 | 5.4 | 58 | -67 | 2 |
|  |  | Hippocampus | 253 | 4.9 | 36 | -14 | -9 |
|  | Insula | |  |  |  |  |  |
|  |  | Insula | 1507 | 7.5 | 36 | 15 | -10 |
|  | Occipital | |  |  |  |  |  |
|  |  | Middle occipital gyrus | 1315 | 5.8 | 43 | -76 | 32 |
|  |  | Inferior occipital gyrus | 360 | 5.6 | 40 | -89 | -4 |
|  |  | Middle occipital gyrus | 300 | 5.6 | 30 | -98 | 2 |
| **Cerebellum** | | |  |  |  |  |  |
|  | Left hemisphere | |  |  |  |  |  |
|  |  | Crus II of cerebellar hemisphere | 743 | 6.2 | -14 | -80 | -44 |
|  |  | Crus I of cerebellar hemisphere | 493 | 6.0 | -41 | -84 | -22 |
|  | Right hemisphere | |  |  |  |  |  |
|  |  | Crus I of cerebellar hemisphere | 596 | 5.1 | 19 | -71 | -36 |

*Size: cluster size (mm3); T: T score of peak; x. y. z: MNI coordinates of peak*

**Table 5.** Clusters identified by the voxel-to-voxel analyses for the linear regression analyses between **peripherical pulse pressure** and brain glycolytic metabolism in the 41-60-year group (p-voxel= 0.005, FWE uncorrected, corrected for the cluster volume).

| **Localization** | | | **size** | **T** | **x** | **y** | **z** |
| --- | --- | --- | --- | --- | --- | --- | --- |
| **Left cerebrum** | | |  |  |  |  |  |
|  | Frontal | |  |  |  |  |  |
|  |  | Superior frontal gyrus | 1236 | 7.5 | -16 | 68 | 7 |
|  |  | Inferior frontal gyrus, triangular part | 617 | 6.7 | -43 | 34 | 25 |
|  |  | Middle frontal gyrus | 600 | 5.7 | -43 | 53 | -7 |
|  |  | Middle frontal gyrus | 304 | 6.0 | -44 | 12 | 45 |
|  |  | Anterior cingulate cortex, supracallosal | 230 | 4.0 | -9 | 35 | 14 |
|  |  | Precentral gyrus | 198 | 4.6 | -53 | 6 | 24 |
|  |  | Precentral gyrus | 114 | 4.9 | -53 | 0 | 38 |
|  | Parietal | |  |  |  |  |  |
|  |  | Middle cingulate & paracingulate gyri | 1024 | 8.7 | -8 | -27 | 37 |
|  |  | Precuneus | 243 | 4.1 | -12 | -59 | 53 |
|  |  | Angular gyrus | 191 | 4.9 | -40 | -55 | 41 |
|  |  | Superior parietal gyrus | 153 | 5.7 | -26 | -53 | 58 |
|  |  | Supramarginal gyrus | 101 | 4.5 | -50 | -39 | 34 |
|  | Temporal | |  |  |  |  |  |
|  |  | Olfactory cortex | 546 | 5.5 | -9 | 8 | -15 |
|  |  | Rolandic operculum | 542 | 6.9 | -60 | 6 | 3 |
|  |  | Middle temporal gyrus | 262 | 4.2 | -61 | -40 | 8 |
|  |  | Rolandic operculum | 237 | 5.3 | 51 | 7 | -1 |
|  |  | Fusiform gyrus | 210 | 6.5 | -28 | -48 | -15 |
|  |  | Middle temporal gyrus | 138 | 3.7 | -65 | -27 | 2 |
|  | Insula | |  |  |  |  |  |
|  |  | Insula | 2828 | 7.7 | -37 | -8 | 4 |
|  | Occipital | |  |  |  |  |  |
|  |  | Middle occipital gyrus | 214 | 6.7 | -47 | -76 | 32 |
|  |  | Superior occipital gyrus | 99 | 4.3 | -10 | -102 | 10 |
| **Right cerebrum** | | |  |  |  |  |  |
|  | Frontal | |  |  |  |  |  |
|  |  | Superior frontal gyrus | 1877 | 8.0 | 30 | 44 | 19 |
|  |  | Middle frontal gyrus | 737 | 6.8 | 38 | 51 | -2 |
|  |  | Inferior frontal gyrus, opercular part | 737 | 5.6 | 50 | 8 | 14 |
|  |  | Precentral gyrus | 406 | 6.8 | 46 | 5 | 42 |
|  |  | Superior frontal gyrus | 256 | 4.9 | 14 | 57 | 26 |
|  |  | Precentral gyrus | 196 | 5.1 | 55 | 3 | 31 |
|  |  | Superior frontal gyrus | 186 | 4.3 | 17 | 0 | 62 |
|  |  | Anterior cingulate cortex, pregenual | 144 | 3.8 | 9 | 37 | 14 |
|  | Parietal | |  |  |  |  |  |
|  |  | Middle cingulate & paracingulate gyri | 2013 | 5.7 | 4 | 1 | 38 |
|  |  | Supramarginal gyrus | 1581 | 6.8 | 58 | -29 | 34 |
|  |  | Angular gyrus | 259 | 4.1 | 55 | -66 | 28 |
|  |  | Angular gyrus | 248 | 4.8 | 59 | -58 | 34 |
|  |  | Precuneus | 236 | 5.7 | 11 | -65 | 48 |
|  |  | Middle cingulate & paracingulate gyri | 201 | 4.8 | 7 | -36 | 34 |
|  |  | Postcentral gyrus | 135 | 4.4 | 30 | -46 | 59 |
|  |  | Postcentral gyrus | 95 | 4.2 | 60 | -11 | 24 |
|  | Temporal | |  |  |  |  |  |
|  |  | Superior temporal gyrus | 419 | 6.1 | 69 | -32 | 8 |
|  |  | Middle temporal gyrus | 347 | 5.0 | 56 | -70 | 2 |
|  |  | Middle temporal gyrus | 263 | 4.9 | 52 | 0 | -19 |
|  |  | Inferior temporal gyrus | 145 | 5.8 | 61 | -59 | -18 |
|  | Basal ganglia | |  |  |  |  |  |
|  |  | Lenticular nucleus, Putamen | 108 | 5.0 | 36 | -15 | -8 |
|  | Occipital | |  |  |  |  |  |
|  |  | Middle occipital gyrus | 451 | 5.7 | 46 | -82 | 20 |
|  |  | Middle occipital gyrus | 195 | 6.0 | 29 | -99 | 6 |
|  |  | Middle occipital gyrus | 178 | 6.9 | 42 | -78 | 6 |
| **Cerebellum** | | |  |  |  |  |  |
|  | Left hemisphere | |  |  |  |  |  |
|  |  | Crus I of cerebellar hemisphere | 557 | 5.6 | -32 | -85 | -22 |
|  |  | Lobule VI of cerebellar hemisphere | 129 | 5.1 | -8 | -81 | -14 |
|  |  |  |  |  |  |  |  |

*Size: cluster size (mm3); T: T score of peak; x. y. z: MNI coordinates of peak*

**Table 6.** Clusters identified by the voxel-to-voxel analyses for the linear regression analyses between **peripherical systolic blood pressure** and brain glycolytic metabolism in the 41-60-year group (p-voxel= 0.005, FWE uncorrected, corrected for the cluster volume).

| **Localization** | | | **size** | **T** | **x** | **y** | **z** |
| --- | --- | --- | --- | --- | --- | --- | --- |
| **Left cerebrum** | | |  |  |  |  |  |
|  | Frontal | |  |  |  |  |  |
|  |  | Superior frontal gyrus | 680 | 6.1 | -20 | 62 | 15 |
|  |  | Inferior frontal gyrus, triangular part | 204 | 6.1 | -43 | 33 | 25 |
|  |  | Anterior cingulate cortex, supracallosal | 191 | 4.1 | 1 | 36 | 5 |
|  |  | Middle frontal gyrus | 173 | 4.4 | -41 | 55 | -7 |
|  |  | Inferior frontal gyrus, triangular part | 126 | 4.2 | -54 | 22 | 9 |
|  |  | Precentral gyrus | 102 | 4.4 | -55 | 6 | 24 |
|  | Parietal | |  |  |  |  |  |
|  |  | Middle cingulate & paracingulate gyri | 866 | 7.4 | -12 | -34 | 37 |
|  |  | Inferior parietal gyrus | 191 | 4.9 | -39 | -54 | 39 |
|  |  | Supramarginal gyrus | 107 | 4.3 | -43 | -42 | 34 |
|  | Temporal | |  |  |  |  |  |
|  |  | Middle temporal gyrus | 597 | 5.0 | -66 | -26 | 2 |
|  |  | Parahippocampal gyrus | 246 | 4.4 | -16 | -5 | -32 |
|  |  | Superior temporal gyrus | 196 | 4.9 | -45 | -25 | 4 |
|  |  | Rolandic operculum | 156 | 5.5 | -60 | 7 | 4 |
|  |  | Fusiform gyrus | 146 | 5.6 | -27 | -49 | -15 |
|  |  | Superior temporal gyrus | 100 | 4.2 | -58 | 0 | -5 |
|  | Insula | |  |  |  |  |  |
|  |  | Insula | 2785 | 9.8 | -38 | -14 | 2 |
|  | Basal ganglia | |  |  |  |  |  |
|  |  | Nucleus accumbens | 394 | 5.4 | -5 | 11 | -2 |
| **Right cerebrum** | | |  |  |  |  |  |
|  | Frontal | |  |  |  |  |  |
|  |  | Inferior frontal gyrus, opercular part | 623 | 5.6 | 51 | 8 | 15 |
|  |  | Middle frontal gyrus | 456 | 6.8 | 41 | 44 | 19 |
|  |  | Precentral gyrus | 430 | 5.3 | 46 | 3 | 44 |
|  |  | Precentral gyrus | 274 | 6.5 | 55 | 4 | 31 |
|  |  | Middle frontal gyrus | 117 | 4.1 | 31 | 35 | 30 |
|  | Parietal | |  |  |  |  |  |
|  |  | Supramarginal gyrus | 2953 | 7.7 | 57 | -26 | 32 |
|  |  | Middle cingulate & paracingulate gyri | 335 | 4.0 | 3 | -18 | 34 |
|  |  | Superior parietal gyrus | 200 | 5.7 | 30 | -47 | 58 |
|  |  | Precuneus | 105 | 4.6 | 12 | -65 | 48 |
|  | Temporal | |  |  |  |  |  |
|  |  | Middle temporal gyrus | 551 | 5.8 | 56 | -69 | 2 |
|  |  | Superior temporal gyrus | 514 | 6.3 | 66 | -29 | 7 |
|  | Basal ganglia | |  |  |  |  |  |
|  |  | Lenticular nucleus, Putamen | 825 | 5.0 | 31 | 12 | 1 |
|  | Occipital | |  |  |  |  |  |
|  |  | Middle occipital gyrus | 442 | 6.5 | 43 | -76 | 33 |
|  |  | Middle occipital gyrus | 281 | 5.6 | 44 | -82 | 24 |
| **Cerebellum** | | |  |  |  |  |  |
|  | Left hemisphere | |  |  |  |  |  |
|  |  | Crus I of cerebellar hemisphere | 599 | 5.0 | -40 | -83 | -22 |
|  |  | Crus II of cerebellar hemisphere | 168 | 4.7 | -16 | -79 | -46 |
|  |  | Lobule VIII of cerebellar hemisphere | 98 | 4.6 | -28 | -36 | -45 |

*Size: cluster size (mm3); T: T score of peak; x. y. z: MNI coordinates of peak*

**Table 7.** Clusters identified by the voxel-to-voxel analyses for the linear regression analyses between **central diastolic blood pressure** and brain glycolytic metabolism in the > 60 year group (p-voxel= 0.005, FWE uncorrected, corrected for the cluster volume).

| **Localization** | | | **size** | **T** | **x** | **y** | **z** |
| --- | --- | --- | --- | --- | --- | --- | --- |
| **Left cerebrum** | | |  |  |  |  |  |
|  | Frontal | |  |  |  |  |  |
|  |  | Superior frontal gyrus | 1683 | 3.9 | -40 | 56 | 24 |
|  |  | Paracentral lobule | 750 | 4.1 | -4 | -35 | 59 |
|  |  | Precentral gyrus | 412 | 3.6 | -58 | 4 | 42 |
|  |  | Gyrus rectus | 390 | 4.2 | -3 | 50 | -15 |
|  |  | Superior frontal gyrus | 297 | 3.6 | -30 | 65 | 10 |
|  |  | Middle frontal gyrus | 237 | 4.2 | -27 | 25 | 46 |
|  |  | Middle frontal gyrus | 193 | 3.3 | 45 | -2 | 60 |
|  |  | Paracentral lobule | 183 | 3.8 | -3 | -24 | 52 |
|  | Temporal | |  |  |  |  |  |
|  |  | Middle temporal gyrus | 2327 | 5.1 | -58 | 1 | -41 |
|  |  | Temporal pole: superior temporal gyrus | 165 | 3.3 | -42 | 4 | -19 |
|  | Occipital | |  |  |  |  |  |
|  |  | Middle occipital gyrus | 364 | 4.9 | -39 | -82 | 3 |
|  | Basal ganglia | |  |  |  |  |  |
|  |  | Medial Geniculate | 898 | 3.6 | -16 | -24 | -7 |
|  |  | Lenticular nucleus, Putamen | 592 | 3.9 | -25 | 8 | -10 |
| **Right cerebrum** | | |  |  |  |  |  |
|  | Frontal | |  |  |  |  |  |
|  |  | Superior frontal gyrus | 3672 | 5.3 | 31 | 9 | 64 |
|  |  | Superior frontal gyrus | 1877 | 4.6 | 21 | 54 | 37 |
|  |  | Superior frontal gyrus | 527 | 3.7 | 29 | 61 | 20 |
|  |  | Inferior frontal gyrus, triangular part | 406 | 3.8 | 55 | 24 | 19 |
|  | Parietal | |  |  |  |  |  |
|  |  | Postcentral gyrus | 285 | 3.9 | 61 | -3 | 38 |
|  | Temporal | |  |  |  |  |  |
|  |  | Middle temporal gyrus | 454 | 4.7 | 70 | -17 | -12 |
|  | Basal ganglia | |  |  |  |  |  |
|  |  | Red nucleus | 349 | 4.4 | 3 | -19 | -13 |
|  |  | Lenticular nucleus, Putamen | 251 | 3.4 | 20 | 12 | -9 |
| **Cerebellum** | | |  |  |  |  |  |
|  | Left hemisphere | |  |  |  |  |  |
|  |  | Lobule VIII of cerebellar hemisphere | 1685 | 4.5 | -29 | -47 | -44 |
|  |  | Crus I of cerebellar hemisphere | 1445 | 5.7 | -58 | -46 | -39 |
|  | Right hemisphere | |  |  |  |  |  |
|  |  | Lobule IX of cerebellar hemisphere | 522 | 3.8 | 11 | -52 | -36 |
|  |  | Crus I of cerebellar hemisphere | 457 | 3.5 | 51 | -41 | -35 |
|  |  | Crus I of cerebellar hemisphere | 307 | 3.7 | 49 | -79 | -29 |

*Size: cluster size (mm3); T: T score of peak; x. y. z: MNI coordinates of peak*

**Table 8.** Clusters identified by the voxel-to-voxel analyses for the linear regression analyses between **heart reate** and brain glycolytic metabolism in the > 60 year group (p-voxel= 0.005, FWE uncorrected, corrected for the cluster volume).

| **Localization** | | | **size** | **T** | **x** | **y** | **z** |
| --- | --- | --- | --- | --- | --- | --- | --- |
| **Left cerebrum** | | |  |  |  |  |  |
|  | Frontal | |  |  |  |  |  |
|  |  | Inferior frontal gyrus, triangular part | 1634 | 4.9 | -48 | 25 | -2 |
|  |  | Superior frontal gyrus | 410 | 4.7 | -23 | 32 | 43 |
|  |  | Anterior cingulate cortex, pregenual | 392 | 3.7 | -5 | 43 | -2 |
|  |  | Middle frontal gyrus | 300 | 4.0 | -44 | 26 | 31 |
|  |  | Gyrus rectus | 293 | 3.6 | -7 | 24 | -23 |
|  |  | Middle frontal gyrus | 215 | 4.3 | -40 | 17 | 49 |
|  |  | Superior frontal gyrus | 194 | 4.4 | -20 | 19 | 60 |
|  | Parietal | |  |  |  |  |  |
|  |  | Supramarginal gyrus | 568 | 3.9 | -62 | -39 | 39 |
|  |  | Angular gyrus | 566 | 4.8 | -47 | -66 | 46 |
|  |  | Precuneus | 329 | 3.2 | -4 | -60 | 34 |
|  |  | Angular gyrus | 183 | 3.3 | -39 | -65 | 27 |
|  |  | Postcentral gyrus | 175 | 3.7 | -35 | -37 | 64 |
|  | Temporal | |  |  |  |  |  |
|  |  | Fusiform gyrus | 1631 | 4.5 | -42 | -67 | -17 |
|  |  | Inferior temporal gyrus | 426 | 3.7 | -60 | -54 | -11 |
|  |  | Temporal pole: middle temporal gyrus | 197 | 3.7 | -44 | 17 | -27 |
|  | Occipital | |  |  |  |  |  |
|  |  | Lingual gyrus | 1968 | 4.3 | -19 | -89 | -17 |
|  |  | Lingual gyrus | 228 | 3.4 | -22 | -61 | -10 |
|  |  | Superior occipital gyrus | 227 | 4.1 | -25 | -91 | 30 |
| **Right cerebrum** | | |  |  |  |  |  |
|  | Frontal | |  |  |  |  |  |
|  |  | Middle frontal gyrus | 472 | 5.2 | 31 | 38 | 35 |
|  |  | Middle frontal gyrus | 467 | 3.8 | 33 | 52 | 14 |
|  |  | Middle frontal gyrus | 422 | 4.4 | 45 | 17 | 45 |
|  |  | Inferior frontal gyrus, triangular part | 257 | 3.8 | 53 | 24 | 18 |
|  |  | Middle frontal gyrus | 256 | 4.3 | 37 | 10 | 53 |
|  | Parietal | |  |  |  |  |  |
|  |  | Angular gyrus | 799 | 4.1 | 60 | -57 | 33 |
|  |  | Inferior parietal gyrus | 359 | 5.1 | 44 | -40 | 49 |
|  |  | Postcentral gyrus | 192 | 4.3 | 59 | -4 | 34 |
|  | Temporal | |  |  |  |  |  |
|  |  | Olfactory cortex | 623 | 4.6 | 20 | 9 | -14 |
|  |  | Superior temporal gyrus | 296 | 4.1 | 65 | -16 | 5 |
|  | Occipital | |  |  |  |  |  |
|  |  | Inferior occipital gyrus | 336 | 4.4 | 27 | -85 | -16 |
|  |  | Lingual gyrus | 174 | 3.9 | 14 | -80 | -13 |
| **Cerebellum** | | |  |  |  |  |  |
|  | Left hemisphere | |  |  |  |  |  |
|  |  | Crus II of cerebellar hemisphere | 1652 | 4.2 | -17 | -88 | -36 |
|  | Right hemisphere | |  |  |  |  |  |
|  |  | Crus II of cerebellar hemisphere | 881 | 4.1 | 37 | -88 | -34 |

*Size: cluster size (mm3); T: T score of peak; x. y. z: MNI coordinates of peak*

**Table 9.** Clusters identified by the voxel-to-voxel analyses for the linear regression analyses between **peripherical diastolic blood pressure** and brain glycolytic metabolism in the > 60 year group (p-voxel= 0.005, FWE uncorrected, corrected for the cluster volume).

| **Localization** | | | **size** | **T** | **x** | **y** | **z** |
| --- | --- | --- | --- | --- | --- | --- | --- |
| **Left cerebrum** | | |  |  |  |  |  |
|  | Frontal | |  |  |  |  |  |
|  |  | Superior frontal gyrus | 1484 | 3.6 | -18 | 67 | 14 |
|  |  | Paracentral lobule | 1470 | 5.1 | -5 | -37 | 62 |
|  |  | Middle frontal gyrus | 360 | 3.4 | 42 | 39 | 36 |
|  |  | Middle frontal gyrus | 268 | 3.3 | -31 | 42 | 45 |
|  |  | Middle frontal gyrus | 166 | 3.6 | 46 | 20 | 49 |
|  | Parietal | |  |  |  |  |  |
|  |  | Temporal pole: Middle temporal gyrus | 1699 | 4.1 | -62 | 8 | -25 |
|  |  | Inferior temporal gyrus | 457 | 4.1 | -31 | 11 | -33 |
|  |  | Temporal pole: superior temporal gyrus | 426 | 3.8 | -43 | 1 | -18 |
|  | Occipital | |  |  |  |  |  |
|  |  | Middle occipital gyrus | 330 | 4.9 | -39 | -82 | 4 |
|  | Basal ganglia | |  |  |  |  |  |
|  |  | Medial Geniculate thalamus | 281 | 3.3 | -16 | -24 | -8 |
|  |  | Lenticular nucleus, Putamen | 248 | 3.9 | 22 | 12 | -7 |
| **Right cerebrum** | | |  |  |  |  |  |
|  | Frontal | |  |  |  |  |  |
|  |  | Superior frontal gyrus | 872 | 3.9 | 31 | 60 | 19 |
|  |  | Supplementary motor area | 828 | 3.6 | 4 | 0 | 73 |
|  |  | Superior frontal gyrus | 778 | 4.6 | 18 | 55 | 38 |
|  |  | Superior frontal gyrus | 654 | 5.4 | 29 | 10 | 63 |
|  |  | Precentral gyrus | 321 | 4.1 | 60 | -2 | 39 |
|  |  | Supplementary motor area | 192 | 3.4 | 9 | 26 | 66 |
|  | Temporal | |  |  |  |  |  |
|  |  | Temporal pole: middle temporal gyrus | 523 | 4.4 | 55 | 17 | -25 |
|  |  | Middle temporal gyrus | 202 | 3.5 | 70 | -16 | -11 |
| **Cerebellum** | | |  |  |  |  |  |
|  | Left hemisphere | |  |  |  |  |  |
|  |  | Lobule VIII of cerebellar hemisphere | 1618 | 4.8 | -29 | -47 | -44 |
|  |  | Crus I of cerebellar hemisphere | 1395 | 4.5 | -60 | -50 | -39 |
|  |  | Lobule VI of cerebellar hemisphere | 195 | 3.7 | -10 | -72 | -12 |
|  | Right hemisphere | |  |  |  |  |  |
|  |  | Lobule X of cerebellar hemisphere | 570 | 4.1 | 24 | -41 | -41 |
|  |  | Crus II of cerebellar hemisphere | 177 | 3.4 | 48 | -41 | -42 |

*Size: cluster size (mm3); T: T score of peak; x. y. z: MNI coordinates of peak*
