# Supplementary figures and images for: Interactions Between Brain 18F-FDG PET Metabolism and Hemodynamic Parameters at Different Ages of Life: Results From a Prospective Cross-Sectional Study
Source: Front Aging Neurosci. 2022 Jun 28;14:908063. doi: 10.3389/fnagi.2022.908063 (PMC9273887; doi:10.3389/fnagi.2022.908063)

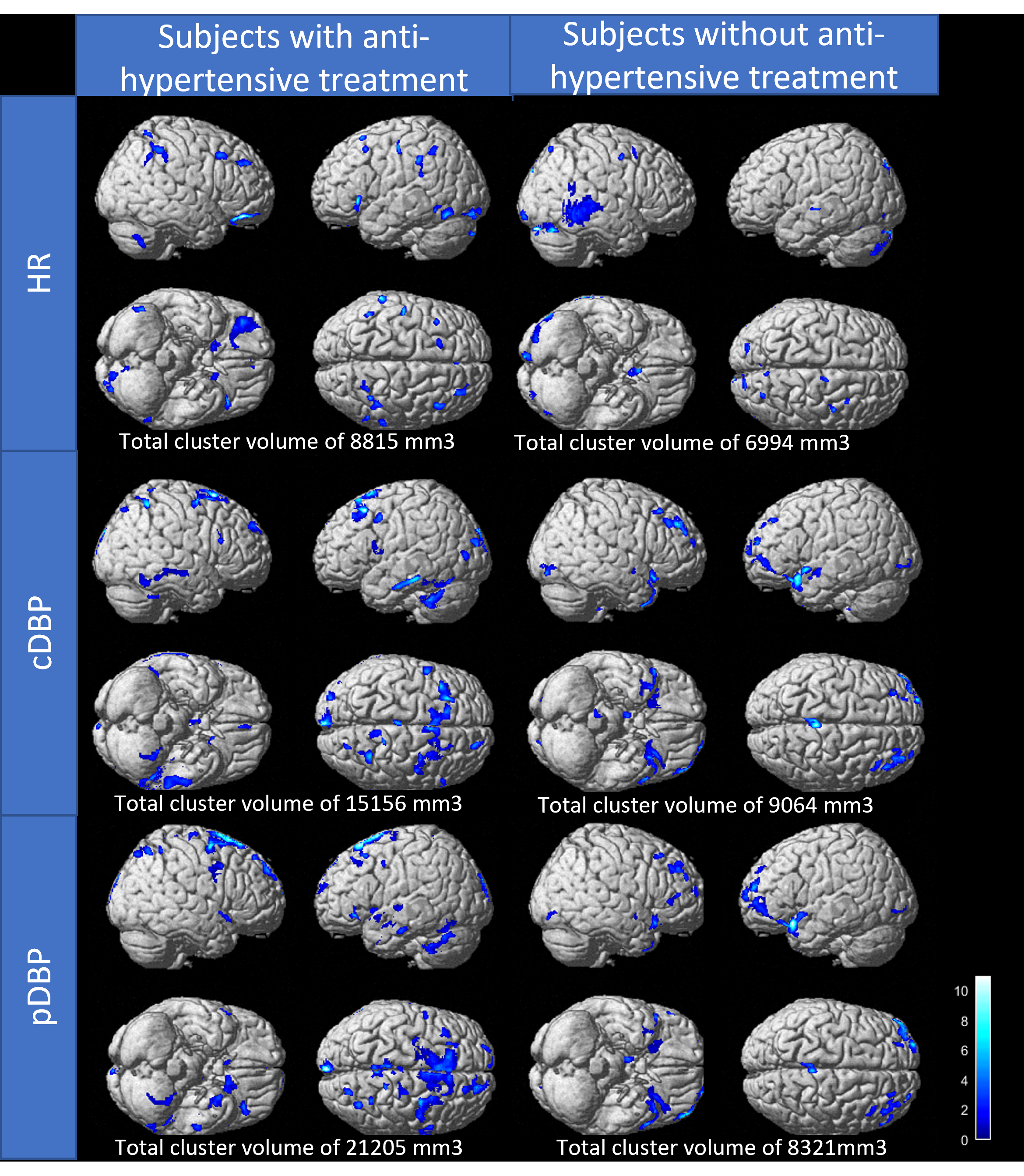

Supplement: Supplementary file 2 [file Image_1.PNG]
